# Methyl 2-(*N*-methoxy­carbonyl­meth­yl-*N*-methylsulfamo­yl)benzoate

**DOI:** 10.1107/S1600536809003742

**Published:** 2009-02-04

**Authors:** Naeem Ahmad, M. Nawaz Tahir, Durre Shahwar, Muhammad Akmal Khan, Uzma Sana

**Affiliations:** a Department of Chemistry, Government College University, Lahore, Pakistan; b Department of Physics, University of Sargodha, Sargodha, Pakistan

## Abstract

In the title compound, C_12_H_15_NO_6_S, the aromatic ring is oriented at dihedral angles of 64.76 (11) and 56.42 (13)° with respect to the planar methyl ester unit and the SO_2_ group, respectively. The dihedral angle between the SO_2_ group and the planar methoxy­carbonyl­methyl group is 50.42 (14)°. Intra­molecular C—H⋯O hydrogen bonding results in the formation of an eight-membered ring. In the crystal structure, inter­molecular C—H⋯O hydrogen bonds link the mol­ecules.

## Related literature

For general background, see: Hanson *et al.* (1999 ▶). For related structures, see: Arshad *et al.* (2008 ▶); Shafiq *et al.* (2008*a* ▶,*b* ▶); Ma *et al.*, 2003 ▶).
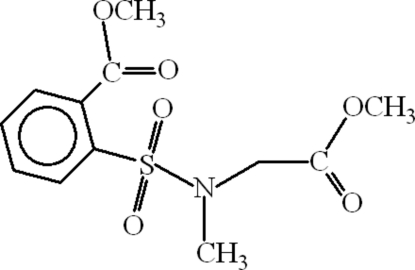


## Experimental

### 

#### Crystal data

C_12_H_15_NO_6_S
*M* *_r_* = 301.31
Orthorhombic, 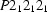

*a* = 8.5830 (3) Å
*b* = 9.0966 (3) Å
*c* = 18.3329 (7) Å
*V* = 1431.36 (9) Å^3^
*Z* = 4
Mo *K*α radiation
μ = 0.25 mm^−1^
*T* = 296 (2) K
0.22 × 0.18 × 0.15 mm

#### Data collection

Bruker Kappa APEXII CCD diffractometer
Absorption correction: multi-scan (*SADABS*; Bruker, 2005 ▶) *T*_min_ = 0.942, *T*_max_ = 0.965
16627 measured reflections
3558 independent reflections
2513 reflections with *I* > 2σ(*I*)
*R*_int_ = 0.039

#### Refinement

*R*[*F* ^2^ > 2σ(*F* ^2^)] = 0.040
*wR*(*F* ^2^) = 0.098
*S* = 1.01
3558 reflections
184 parameters
H-atom parameters constrained
Δρ_max_ = 0.16 e Å^−3^
Δρ_min_ = −0.24 e Å^−3^
Absolute structure: Flack (1983 ▶), 1509 Friedel pairs
Flack parameter: 0.12 (8)

### 

Data collection: *APEX2* (Bruker, 2007 ▶); cell refinement: *SAINT* (Bruker, 2007 ▶); data reduction: *SAINT*; program(s) used to solve structure: *SHELXS97* (Sheldrick, 2008 ▶); program(s) used to refine structure: *SHELXL97* (Sheldrick, 2008 ▶); molecular graphics: *ORTEP-3 for Windows* (Farrugia, 1997 ▶) and *PLATON* (Spek, 2003 ▶); software used to prepare material for publication: *WinGX* (Farrugia, 1999 ▶) and *PLATON*.

## Supplementary Material

Crystal structure: contains datablocks global, I. DOI: 10.1107/S1600536809003742/hk2618sup1.cif

Structure factors: contains datablocks I. DOI: 10.1107/S1600536809003742/hk2618Isup2.hkl

Additional supplementary materials:  crystallographic information; 3D view; checkCIF report

## Figures and Tables

**Table 1:** Hydrogen-bond geometry (Å, °)

*D*—H⋯*A*	*D*—H	H⋯*A*	*D*⋯*A*	*D*—H⋯*A*
C9—H9*A*⋯O1	0.97	2.20	3.125 (3)	159
C9—H9*B*⋯O1^i^	0.97	2.56	3.321 (3)	135
C12—H12*B*⋯O4^ii^	0.96	2.44	3.041 (4)	120

Symmetry codes: (i) 
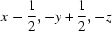
; (ii) 
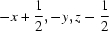
.

**Fig. 1.**
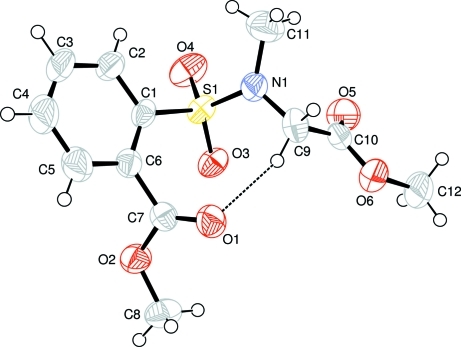
The molecular structure of the title molecule, with the atom-numbering scheme. Displacement ellipsoids are drawn at the 50% probability level. Hydrogen bond is shown as dotted line.

**Fig. 2.**
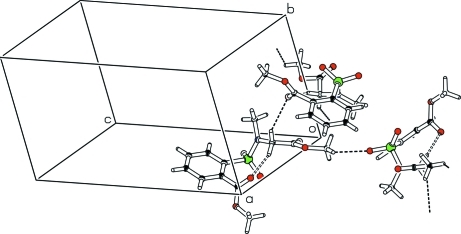
A partial packing diagram of the title compound. Hydrogen bonds are shown as dashed lines.

## supplementary crystallographic information

### Comment

Sulfonamides are a class of compounds, which find wide applications in medicinal chemistry (Hanson *et al.*, 1999). Cyclic sulfonamides (benzothiazine) have biological activities such as lipoxygenase inhibition, and they are used as drugs for heart diseases. We are engaged in the syntheses of various derivatives of benzothiazine molecule (Arshad *et al.*, 2008; Shafiq *et al.*, 20082008*a*,*b*). We report herein the crystal structure of the title compound, (I), which is used as an intermediate for further syntheses.

In the molecule of the title compound, (I), (Fig. 1), the coordination around the S atom is a distorted tetrahedral. The crystal structure of methyl 2-(4-methoxypyrimidin-2-ylcarbamoylsulfamoyl)benzoate, (II) (Ma *et al.*, 2003) has been reported, which also has a sulfamoylbenzoate moiety. In (I), the benzene ring A (C1-C6) is oriented with respect to the planar methyl ester moiety (O1/O2/C7/C8) and SO_2_ group at dihedral angles of 64.76 (11)° and 56.42 (13)°, respectively. The dihedral angle between SO_2_ moiety and the planar methoxycarbonylmethyl group (O5/O6/N1/C9/C10/C12) is 50.42 (14)°. Intramolecular C—H···O hydrogen bonding (Table 1) results in the formation of an eight-membered ring (S1/O1/N1/C1/C6/C7/C9/H9A).

In the crystal structure, intermolecular C—H···O hydrogen bonds (Table 1) link the molecules (Fig. 2), in which they may be effective in the stabilization of the structure.

### Experimental

For the preparation of the title compound, sodium saccharine (20.5 g, 0.1 mol) and methylchloroacetic acid (10.85 g, 0.1 mol) were dissolved in DMF (50 ml) and refluxed for 1 h, and then ice was added for precipitation. The precipitate (12.8 g, 0.05 mol) was dissolved in methanol (50 ml), and sodium methoxide (5.4 g, 0.1 mol) was added, and then refluxed for 3 h. The volume was reduced to half by evaporation. Then, HCl was added on cooling and left overnight in refrigerator, which was then recrystallized from absolute ethanol.

### Refinement

H atoms were positioned geometrically, with C-H = 0.93, 0.96 and 0.97 Å for aromatic, methyl and methylene group and constrained to ride on their parent atoms with U_iso_(H) = xU_eq_(C), where x = 1.5 for methyl H, and x = 1.2 for all other H atoms.

### Crystal data

**Table:**

C_12_H_15_NO_6_S	*F*(000) = 632
*M**_r_* = 301.31	*D*_x_ = 1.398 Mg m^−^^3^
Orthorhombic, *P*2_1_2_1_2_1_	Mo *K*α radiation, λ = 0.71073 Å
Hall symbol: P 2ac 2ab	Cell parameters from 3558 reflections
*a* = 8.5830 (3) Å	θ = 2.2–28.3°
*b* = 9.0966 (3) Å	µ = 0.25 mm^−^^1^
*c* = 18.3329 (7) Å	*T* = 296 K
*V* = 1431.36 (9) Å^3^	Prism, colorless
*Z* = 4	0.22 × 0.18 × 0.15 mm

### Data collection

**Table:**

Bruker Kappa APEXII CCD diffractometer	3558 independent reflections
Radiation source: fine-focus sealed tube	2513 reflections with *I* > 2σ(*I*)
graphite	*R*_int_ = 0.039
Detector resolution: 7.40 pixels mm^-1^	θ_max_ = 28.3°, θ_min_ = 2.2°
ω scans	*h* = −11→7
Absorption correction: multi-scan (*SADABS*; Bruker, 2005)	*k* = −12→10
*T*_min_ = 0.942, *T*_max_ = 0.965	*l* = −24→24
16627 measured reflections	

### Refinement

**Table:**

Refinement on *F*^2^	Secondary atom site location: difference Fourier map
Least-squares matrix: full	Hydrogen site location: inferred from neighbouring sites
*R*[*F*^2^ > 2σ(*F*^2^)] = 0.040	H-atom parameters constrained
*wR*(*F*^2^) = 0.098	*w* = 1/[σ^2^(*F*_o_^2^) + (0.0476*P*)^2^ + 0.0688*P*] where *P* = (*F*_o_^2^ + 2*F*_c_^2^)/3
*S* = 1.01	(Δ/σ)_max_ < 0.001
3558 reflections	Δρ_max_ = 0.16 e Å^−^^3^
184 parameters	Δρ_min_ = −0.24 e Å^−^^3^
0 restraints	Absolute structure: Flack (1983), 1509 Friedel pairs
Primary atom site location: structure-invariant direct methods	Flack parameter: 0.12 (8)

### Special details

**Table:**

Geometry. Bond distances, angles *etc*. have been calculated using the rounded fractional coordinates. All su's are estimated from the variances of the (full) variance-covariance matrix. The cell e.s.d.'s are taken into account in the estimation of distances, angles and torsion angles
Refinement. Refinement of *F*^2^ against ALL reflections. The weighted *R*-factor *wR* and goodness of fit *S* are based on *F*^2^, conventional *R*-factors *R* are based on *F*, with *F* set to zero for negative *F*^2^. The threshold expression of *F*^2^ > σ(*F*^2^) is used only for calculating *R*-factors(gt) *etc*. and is not relevant to the choice of reflections for refinement. *R*-factors based on *F*^2^ are statistically about twice as large as those based on *F*, and *R*- factors based on ALL data will be even larger.

### Fractional atomic coordinates and isotropic or equivalent isotropic displacement parameters (Å^2^)

**Table:**

	*x*	*y*	*z*	*U*_iso_*/*U*_eq_	
S1	0.48790 (6)	0.03278 (6)	0.16077 (3)	0.0454 (2)	
O1	0.78607 (18)	0.0316 (2)	0.03285 (9)	0.0594 (6)	
O2	0.8987 (2)	−0.15351 (18)	0.09228 (8)	0.0578 (6)	
O3	0.5106 (2)	−0.09093 (17)	0.11432 (8)	0.0528 (5)	
O4	0.3864 (2)	0.0227 (2)	0.22151 (9)	0.0730 (7)	
O5	0.1989 (2)	0.0586 (2)	0.01831 (11)	0.0744 (8)	
O6	0.3568 (3)	0.0937 (2)	−0.07700 (10)	0.0802 (8)	
N1	0.4279 (2)	0.1681 (2)	0.11144 (10)	0.0534 (7)	
C1	0.6735 (3)	0.0822 (2)	0.19630 (11)	0.0437 (7)	
C2	0.6751 (3)	0.1563 (3)	0.26251 (12)	0.0581 (8)	
C3	0.8149 (4)	0.1958 (3)	0.29366 (16)	0.0783 (11)	
C4	0.9518 (4)	0.1598 (3)	0.26066 (17)	0.0883 (12)	
C5	0.9524 (3)	0.0831 (3)	0.19607 (15)	0.0705 (10)	
C6	0.8129 (3)	0.0454 (2)	0.16210 (12)	0.0453 (7)	
C7	0.8263 (2)	−0.0253 (3)	0.08873 (12)	0.0451 (7)	
C8	0.9334 (3)	−0.2224 (3)	0.02298 (14)	0.0691 (10)	
C9	0.4512 (3)	0.1665 (3)	0.03348 (12)	0.0551 (8)	
C10	0.3184 (3)	0.0993 (3)	−0.00666 (13)	0.0524 (8)	
C11	0.3366 (4)	0.2883 (3)	0.14377 (16)	0.0815 (11)	
C12	0.2408 (5)	0.0357 (5)	−0.12639 (17)	0.1163 (16)	
H2	0.58187	0.17913	0.28577	0.0698*	
H3	0.81583	0.24736	0.33747	0.0940*	
H4	1.04565	0.18742	0.28200	0.1058*	
H5	1.04666	0.05609	0.17491	0.0846*	
H8A	0.83789	−0.24343	−0.00223	0.1037*	
H8B	0.98933	−0.31225	0.03123	0.1037*	
H8C	0.99584	−0.15722	−0.00600	0.1037*	
H9A	0.54547	0.11201	0.02250	0.0661*	
H9B	0.46577	0.26658	0.01653	0.0661*	
H11A	0.37475	0.38079	0.12602	0.1224*	
H11B	0.34631	0.28520	0.19591	0.1224*	
H11C	0.22901	0.27731	0.13049	0.1224*	
H12A	0.21196	−0.06164	−0.11128	0.1744*	
H12B	0.28284	0.03215	−0.17488	0.1744*	
H12C	0.15056	0.09800	−0.12580	0.1744*	

### Atomic displacement parameters (Å^2^)

**Table:**

	*U*^11^	*U*^22^	*U*^33^	*U*^12^	*U*^13^	*U*^23^
S1	0.0541 (3)	0.0436 (3)	0.0386 (3)	−0.0130 (3)	0.0029 (3)	−0.0013 (3)
O1	0.0599 (9)	0.0687 (12)	0.0497 (9)	0.0096 (9)	0.0026 (8)	0.0119 (9)
O2	0.0758 (11)	0.0440 (10)	0.0535 (10)	0.0095 (9)	−0.0038 (8)	−0.0040 (8)
O3	0.0687 (10)	0.0402 (8)	0.0495 (8)	−0.0140 (8)	−0.0024 (9)	−0.0063 (7)
O4	0.0796 (11)	0.0836 (13)	0.0558 (10)	−0.0252 (11)	0.0226 (9)	−0.0036 (10)
O5	0.0505 (10)	0.0879 (16)	0.0849 (13)	−0.0031 (10)	−0.0139 (10)	−0.0223 (12)
O6	0.1054 (14)	0.0852 (14)	0.0501 (11)	0.0268 (12)	−0.0149 (10)	−0.0049 (10)
N1	0.0599 (11)	0.0491 (12)	0.0511 (11)	0.0039 (9)	−0.0131 (9)	−0.0068 (10)
C1	0.0611 (13)	0.0340 (12)	0.0360 (11)	−0.0023 (10)	−0.0100 (10)	0.0024 (9)
C2	0.0782 (16)	0.0507 (15)	0.0455 (13)	0.0060 (14)	−0.0108 (12)	−0.0074 (12)
C3	0.100 (2)	0.069 (2)	0.0658 (18)	0.0140 (18)	−0.0382 (17)	−0.0274 (15)
C4	0.084 (2)	0.077 (2)	0.104 (2)	0.0090 (17)	−0.0509 (18)	−0.0333 (19)
C5	0.0603 (15)	0.0640 (18)	0.0873 (19)	0.0066 (13)	−0.0235 (14)	−0.0177 (15)
C6	0.0534 (11)	0.0350 (13)	0.0476 (12)	0.0020 (10)	−0.0107 (11)	−0.0001 (11)
C7	0.0418 (11)	0.0428 (13)	0.0506 (13)	−0.0012 (10)	−0.0020 (10)	0.0018 (11)
C8	0.0825 (18)	0.0591 (18)	0.0657 (16)	0.0050 (14)	0.0069 (14)	−0.0177 (14)
C9	0.0541 (13)	0.0553 (16)	0.0559 (14)	−0.0002 (11)	−0.0089 (11)	0.0131 (12)
C10	0.0553 (14)	0.0493 (14)	0.0526 (15)	0.0177 (12)	−0.0147 (12)	−0.0042 (12)
C11	0.0798 (19)	0.0618 (19)	0.103 (2)	0.0132 (16)	−0.0086 (17)	−0.0203 (17)
C12	0.148 (3)	0.124 (3)	0.077 (2)	0.057 (3)	−0.064 (2)	−0.044 (2)

### Geometric parameters (Å, °)

**Table:**

S1—O3	1.4246 (16)	C6—C7	1.495 (3)
S1—O4	1.4168 (18)	C9—C10	1.488 (4)
S1—N1	1.6119 (19)	C2—H2	0.9300
S1—C1	1.779 (3)	C3—H3	0.9300
O1—C7	1.199 (3)	C4—H4	0.9300
O2—C7	1.323 (3)	C5—H5	0.9300
O2—C8	1.448 (3)	C8—H8A	0.9600
O5—C10	1.183 (3)	C8—H8B	0.9600
O6—C10	1.332 (3)	C8—H8C	0.9600
O6—C12	1.446 (4)	C9—H9A	0.9700
N1—C9	1.443 (3)	C9—H9B	0.9700
N1—C11	1.470 (3)	C11—H11A	0.9600
C1—C2	1.389 (3)	C11—H11B	0.9600
C1—C6	1.392 (3)	C11—H11C	0.9600
C2—C3	1.377 (4)	C12—H12A	0.9600
C3—C4	1.362 (5)	C12—H12B	0.9600
C4—C5	1.374 (4)	C12—H12C	0.9600
C5—C6	1.393 (4)		
			
S1···O1	3.4712 (17)	C9···O1	3.125 (3)
O1···S1	3.4712 (17)	C9···O1^v^	3.321 (3)
O1···O3	3.011 (2)	C9···O5^i^	3.417 (3)
O1···C9	3.125 (3)	C10···O3	3.261 (3)
O1···C9^i^	3.321 (3)	C10···C8^ii^	3.580 (4)
O1···C10^i^	3.403 (3)	C10···O1^v^	3.403 (3)
O3···O1	3.011 (2)	C11···O5	3.325 (3)
O3···C8^ii^	3.108 (3)	C12···O4^x^	3.041 (4)
O3···C7	2.814 (2)	C7···H9A	2.9700
O3···C10	3.261 (3)	C10···H11C	3.0900
O4···C12^iii^	3.041 (4)	C10···H8B^ii^	3.0300
O4···C2^iv^	3.387 (3)	C11···H12C^i^	2.9100
O5···N1	2.788 (3)	H2···O4	2.5000
O5···C11	3.325 (3)	H2···O3^viii^	2.8900
O5···C9^v^	3.417 (3)	H3···O2^xi^	2.9100
O1···H8C	2.5900	H4···O2^xi^	2.7600
O1···H9B^i^	2.5600	H5···O2	2.7500
O1···H8A	2.6200	H8A···O1	2.6200
O1···H9A	2.2000	H8B···O3^ix^	2.8200
O2···H4^vi^	2.7600	H8B···C10^ix^	3.0300
O2···H5	2.7500	H8C···O1	2.5900
O2···H3^vi^	2.9100	H8C···O5^xii^	2.6600
O3···H2^iv^	2.8900	H9A···O1	2.2000
O3···H8B^ii^	2.8200	H9A···O3	2.5200
O3···H9A	2.5200	H9A···C7	2.9700
O4···H2	2.5000	H9B···H11A	2.3900
O4···H11B	2.4600	H9B···O1^v^	2.5600
O4···H12B^iii^	2.4400	H9B···O5^i^	2.6300
O5···H8C^vii^	2.6600	H11A···H9B	2.3900
O5···H12A	2.6200	H11A···H12C^i^	2.3800
O5···H12C	2.7000	H11B···O4	2.4600
O5···H11C	2.8700	H11C···O5	2.8700
O5···H9B^v^	2.6300	H11C···C10	3.0900
N1···O5	2.788 (3)	H12A···O5	2.6200
N1···H12C^i^	2.8700	H12B···O4^x^	2.4400
C2···O4^viii^	3.387 (3)	H12C···O5	2.7000
C7···O3	2.814 (2)	H12C···N1^v^	2.8700
C8···O3^ix^	3.108 (3)	H12C···C11^v^	2.9100
C8···C10^ix^	3.580 (4)	H12C···H11A^v^	2.3800
			
O3—S1—O4	120.18 (10)	C2—C3—H3	120.00
O3—S1—N1	108.15 (10)	C4—C3—H3	120.00
O3—S1—C1	107.22 (10)	C3—C4—H4	120.00
O4—S1—N1	107.09 (10)	C5—C4—H4	120.00
O4—S1—C1	106.21 (10)	C4—C5—H5	120.00
N1—S1—C1	107.37 (9)	C6—C5—H5	120.00
C7—O2—C8	115.79 (18)	O2—C8—H8A	109.00
C10—O6—C12	116.7 (3)	O2—C8—H8B	109.00
S1—N1—C9	120.24 (16)	O2—C8—H8C	109.00
S1—N1—C11	120.81 (16)	H8A—C8—H8B	109.00
C9—N1—C11	118.7 (2)	H8A—C8—H8C	109.00
S1—C1—C2	116.85 (19)	H8B—C8—H8C	110.00
S1—C1—C6	122.97 (16)	N1—C9—H9A	109.00
C2—C1—C6	120.1 (2)	N1—C9—H9B	109.00
C1—C2—C3	119.9 (2)	C10—C9—H9A	109.00
C2—C3—C4	120.3 (3)	C10—C9—H9B	109.00
C3—C4—C5	120.5 (3)	H9A—C9—H9B	108.00
C4—C5—C6	120.5 (3)	N1—C11—H11A	109.00
C1—C6—C5	118.6 (2)	N1—C11—H11B	109.00
C1—C6—C7	125.1 (2)	N1—C11—H11C	109.00
C5—C6—C7	116.2 (2)	H11A—C11—H11B	109.00
O1—C7—O2	123.9 (2)	H11A—C11—H11C	109.00
O1—C7—C6	124.1 (2)	H11B—C11—H11C	109.00
O2—C7—C6	111.78 (18)	O6—C12—H12A	109.00
N1—C9—C10	112.8 (2)	O6—C12—H12B	109.00
O5—C10—O6	125.3 (2)	O6—C12—H12C	109.00
O5—C10—C9	127.0 (2)	H12A—C12—H12B	109.00
O6—C10—C9	107.8 (2)	H12A—C12—H12C	109.00
C1—C2—H2	120.00	H12B—C12—H12C	109.00
C3—C2—H2	120.00		
			
O3—S1—N1—C9	−19.1 (2)	S1—C1—C2—C3	179.0 (2)
O3—S1—N1—C11	155.37 (19)	C6—C1—C2—C3	1.2 (3)
O4—S1—N1—C9	−149.96 (17)	S1—C1—C6—C5	−176.98 (17)
O4—S1—N1—C11	24.5 (2)	S1—C1—C6—C7	6.6 (3)
C1—S1—N1—C9	96.32 (18)	C2—C1—C6—C5	0.7 (3)
C1—S1—N1—C11	−89.2 (2)	C2—C1—C6—C7	−175.8 (2)
O3—S1—C1—C2	−154.78 (17)	C1—C2—C3—C4	−1.4 (4)
O3—S1—C1—C6	22.94 (19)	C2—C3—C4—C5	−0.3 (4)
O4—S1—C1—C2	−25.1 (2)	C3—C4—C5—C6	2.2 (4)
O4—S1—C1—C6	152.60 (17)	C4—C5—C6—C1	−2.4 (4)
N1—S1—C1—C2	89.20 (19)	C4—C5—C6—C7	174.4 (2)
N1—S1—C1—C6	−93.09 (18)	C1—C6—C7—O1	64.8 (3)
C8—O2—C7—O1	2.1 (3)	C1—C6—C7—O2	−120.0 (2)
C8—O2—C7—C6	−173.08 (19)	C5—C6—C7—O1	−111.8 (2)
C12—O6—C10—O5	1.3 (4)	C5—C6—C7—O2	63.4 (3)
C12—O6—C10—C9	−178.1 (3)	N1—C9—C10—O5	5.0 (4)
S1—N1—C9—C10	91.0 (2)	N1—C9—C10—O6	−175.5 (2)
C11—N1—C9—C10	−83.6 (3)		

Symmetry codes: (i) *x*+1/2, −*y*+1/2, −*z*; (ii) *x*−1/2, −*y*−1/2, −*z*; (iii) −*x*+1/2, −*y*, *z*+1/2; (iv) −*x*+1, *y*−1/2, −*z*+1/2; (v) *x*−1/2, −*y*+1/2, −*z*; (vi) −*x*+2, *y*−1/2, −*z*+1/2; (vii) *x*−1, *y*, *z*; (viii) −*x*+1, *y*+1/2, −*z*+1/2; (ix) *x*+1/2, −*y*−1/2, −*z*; (x) −*x*+1/2, −*y*, *z*−1/2; (xi) −*x*+2, *y*+1/2, −*z*+1/2; (xii) *x*+1, *y*, *z*.

### Hydrogen-bond geometry (Å, °)

**Table:**

*D*—H···*A*	*D*—H	H···*A*	*D*···*A*	*D*—H···*A*
C9—H9A···O1	0.97	2.20	3.125 (3)	159
C9—H9B···O1^v^	0.97	2.56	3.321 (3)	135
C12—H12B···O4^x^	0.96	2.44	3.041 (4)	120

Symmetry codes: (v) *x*−1/2, −*y*+1/2, −*z*; (x) −*x*+1/2, −*y*, *z*−1/2.
